# Exploring the Frontier: Antisense Long Non-Coding RNAs as Key Regulators in Alzheimer’s Disease

**DOI:** 10.14336/AD.2024.0762

**Published:** 2025-08-19

**Authors:** Jing Cai, Yu-Qing Ni, You-Shuo Liu

**Affiliations:** ^1^Department of Geriatrics, The Second Xiangya Hospital of Central South University, Changsha, Hunan, China.; ^2^Institute of Aging and Age-related Disease Research, Central South University, Changsha, Hunan, China.

**Keywords:** Alzheimer’s disease;, Antisense long non-coding RNAs, Pathogenesis

## Abstract

Alzheimer's Disease (AD) is the most prevalent, costly, and fatal neurodegenerative disorder of this century. Two hallmark features of AD are the anomalous cleavage of amyloid precursor protein (APP), which leads to the accumulation of amyloid-beta (Aβ), and the hyperphosphorylation of tau protein. Despite extensive research efforts, the pathology and pathogenesis of AD remain elusive. Recent investigations have highlighted the close association between antisense long non-coding RNAs (AS-lncRNAs) and various biological and functional aspects of AD. However, many AS-lncRNAs implicated in AD have not yet been comprehensively compiled and discussed. This paper reviews the role of AS-lncRNAs in neurodegenerative diseases, outlines their association with AD, and offers novel insights into the potential applications of antisense RNAs in the diagnosis and treatment of AD.

## 1. Introduction

Alzheimer’s Disease (AD) is one of the most prevalent neurodegenerative disorders and a leading cause of dementia in the aging population [1]. Currently, this progressive neurodegenerative disease affects approximately 10% of the global population, inflicting indescribable suffering and consuming over $100 billion annually in healthcare costs [2]. Clinically, AD manifests as progressive memory impairment, accompanied by cognitive dysfunction and personality changes, with pathological hallmarks including senile plaques formed by the aggregation of pathogenic amyloid-β (Aβ) protein, neurofibrillary tangles composed of hyperphosphorylated tau within neurons, and neuronal loss [3]. Current research efforts are focused on targeting and modulating neurotransmitter imbalances to alleviate disease symptoms. These approaches include drugs targeting cholinergic or glutamatergic neurotransmission, gene therapy, and immunotherapy [4]. While effective medications can improve symptoms and enhance quality of life, there are currently no drugs that can cure or prevent the progression of cognitive impairment [5].

Long non-coding RNAs (lncRNAs) are a class of RNA transcripts over 200 nucleotides in length, devoid of protein-coding capacity [6]. In cell biology, lncRNAs play crucial roles in various physiological processes, including gene regulation, chromatin remodeling, post-transcriptional regulation, and epigenetic modulation. These RNA molecules interact with DNA, RNA, and proteins to influence gene expression and cellular functions, participating in key processes such as cell differentiation, development, metabolism, and immunity [7]. Previous studies have shown that lncRNAs play diverse roles in key physiological processes such as metabolism and immunity and are closely associated with the development of diseases including cancer, cardiovascular diseases, neurological disorders, and renal diseases [8-10]. Based on their genomic positions relative to protein-coding genes, lncRNAs can be categorized into sense lncRNAs, antisense lncRNAs (AS-lncRNAs), intronic lncRNAs, bidirectional lncRNAs, intergenic lncRNAs, and enhancer lncRNAs [11]. AS-lncRNAs, as one of the categories of lncRNAs, are transcribed from the opposite strand of protein-coding or non-protein-coding genes, serving as counterparts to coding gene sense strands [12]. AS-lncRNAs can interact with DNA, RNA, and proteins, thus sharing the mechanisms of gene regulation known for other lncRNA biotypes, and also rerouting them onto their sense genes. With the rapid development of RNA sequencing (RNA-seq) technology, the dynamic expression patterns and functions of AS-lncRNAs under pathological and physiological conditions and during development have been characterized [13]. Recent studies indicate that abnormal expression and dysregulation of lncRNAs are linked to several facets of AD etiology and pathophysiology, including Aβ peptide accumulation, synaptic dysfunction, and inflammation [14]. Abundant AS-lncRNAs have been discovered in human diseases, particularly in cancers and neurological disorders [15, 16]. However, there is currently no comprehensive review regarding the roles and mechanisms of AS-lncRNAs in AD.

In this review, we summarize the pathological mechanisms underlying AD, introduce the various roles of AS-lncRNAs in the pathogenesis of AD, and elucidate the potential applications of AS-lncRNAs in the diagnosis and treatment of AD-related disorders.

## 2. Role of antisense lncRNAs in Neurodegenerative Diseases

Neurodegenerative diseases are disorders marked by structural damage to the central nervous system and a gradual decline in neurological function [17, 18]. These diseases feature abnormal protein conformations and spatial distributions [19]. Recently, changes in lncRNAs expression have drawn significant attention in neuronal disorders, highlighting their impact on abnormal molecular pathways [20]. LncRNAs, among non-coding genes, are highly expressed in the nervous system and frequently found near genes and sites linked to neurodegenerative diseases [21]. AS-lncRNAs constitute a significant portion of lncRNAs, particularly prevalent in the central nervous system, where they are speculated to regulate crucial neuronal processes [22]. Both in vitro and in vivo studies suggest that targeting AS-lncRNAs could alleviate the severity of neurodegenerative diseases. For instance, inhibiting BDNF-AS has been shown to enhance neuronal survival [23]. Therefore, AS-lncRNAs could be potential therapeutic targets for neurodegenerative diseases.

**Figure 1.**
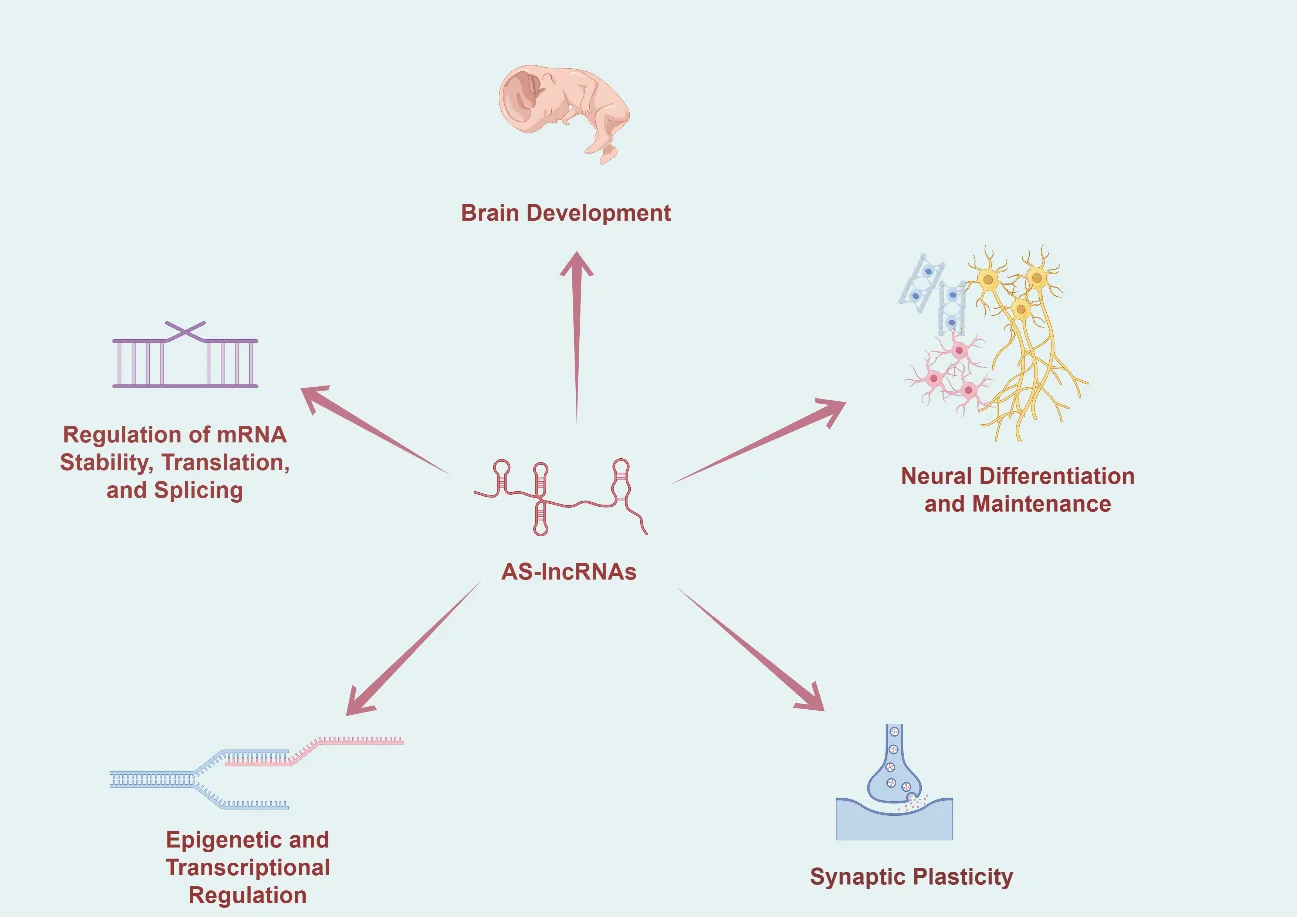
**Role of antisense lncRNA in neurodegenerative diseases**. AS-lncRNAs play a crucial role in brain development, neural differentiation and maintenance, and synaptic plasticity. They also influence the onset and progression of neurodegenerative diseases by participating in epigenetic and transcriptional regulation and by modulating mRNA stability, translation, and splicing.

### 2.1 AS-lncRNAs in Brain Health and Disease

The vertebrate brain, especially in birds and mammals, is among the most complex biological systems. lncRNAs play a crucial role in the central nervous system, with their expression being significantly more brain-specific than in other tissues [24, 25]. Studies indicate that up to 40% of differentially expressed lncRNAs are brain-specific [26]. Multiple transcriptomic studies have revealed that thousands of lncRNAs are expressed during brain development [27-30]. Although lncRNAs generally exhibit low conservation, brain-specific lncRNAs tend to have higher sequence conservation compared to those in other tissues [31, 32]. AS-lncRNAs make up a significant portion of lncRNAs, and the dysregulation of many AS-lncRNAs is linked to neurodegenerative diseases [19]. Additionally, about 70% of genes are transcribed in the antisense direction, generating AS-lncRNA. These AS-lncRNAs play crucial roles in brain development, neuronal differentiation and maintenance, and synaptic plasticity. They also influence neurodegenerative disease development and progression by participating in epigenetic and transcriptional regulation and modulating mRNA stability, translation, or splicing [33-38] (Fig. 1).

### 2.1.1. Role of AS-lncRNAs in Brain Development

The Allen Brain Atlas (ABA) is a large-scale gene expression study that utilizes high-throughput RNA in situ hybridization to visualize the expression of over 20,000 transcripts in the adult mouse brain, with more than 1,000 probes targeting non-coding transcripts [39]. Further analysis of this data revealed that 849 non-coding RNAs (out of 1,328 examined) are expressed in the adult mouse brain, with the majority associated with specific neuroanatomical regions, cell types, or subcellular compartments [40]. This study provides compelling evidence that many of these transcripts are functionally significant.

AS-lncRNAs form a significant part of lncRNAs, regulating the expression of sense transcripts or affecting the processing of sense mRNAs [22]. During cortical development, several AS-lncRNAs are expressed to finely tune the levels of transcription factors [33]. An example is the Sox (SRY-related HMG-box) gene family, which encodes high-mobility group proteins that can bind and bend DNA during embryonic development, thereby influencing cell fate. Specifically, two members of the Sox family, Sox4 and Sox11, have corresponding AS-lncRNAs that are highly expressed during mouse cortical development, suggesting that they may have specific functions in corticogenesis and cell differentiation [41].

### 2.1.2. Application of AS-lncRNAs in Neural Differentiation and Maintenance

LncRNAs play crucial roles in neural development and differentiation. Researchers have identified various evolutionarily conserved lncRNA transcripts that are dynamically and specifically expressed in both developing and mature cell types of the mouse retina, as revealed by gene expression sequence analysis (SAGE) conducted at multiple time points. This indicates that these lncRNAs are vital for neuronal development and physiological functions [22]. Furthermore, an analysis of 169 lncRNAs that are dynamically expressed during neural stem cell-mediated processes demonstrates co-expression with protein-coding genes essential for neural development. This further suggests that lncRNAs share regulatory mechanisms with protein-coding genes and are integrated into intricate gene expression programs involved in neural development [42].

In the central nervous system, AS-lncRNAs are particularly prevalent and are thought to regulate crucial neuronal processes [43]. Among these, BDNF-AS is a notable AS-lncRNA that primarily regulates the levels of brain-derived neurotrophic factor (BDNF). BDNF is associated with several critical physiological processes, including neurogenesis, progenitor cell proliferation, neuronal differentiation, maturation, and plasticity, and plays a significant role in the pathophysiology of neurodegenerative diseases [37]. Overexpression of BDNF-AS results in decreased BDNF levels, both in vitro and in vivo [23]. Although the precise functional mechanisms of BDNF-AS remain unclear, its expression seems to influence the BDNF gene by recruiting EZH2 (a histone methyltransferase), leading to the inactivation of the BDNF promoter. Studies have shown that knocking down BDNF-AS increases the number of neuronal cells, enhances differentiation, and promotes significant neurite growth [23, 44].

Additionally, the Nkx2.2 antisense gene (Nkx2.2 AS) can cis-regulate the transcription levels of Nkx2.2, which is a transcription factor. Upregulation of Nkx2.2 promotes oligodendrocyte differentiation, while overexpression of Nkx2.2 AS induces a moderate increase in Nkx2.2 mRNA levels [45]. Research on retinal development indicates that Six3 opposite strand (Six3OS) can act as a molecular scaffold to recruit histone-modifying enzymes to Six3 target genes, thereby regulating Six3 activity and influencing retinal cell growth [46].

### 2.1.3. Role of AS-lncRNAs in Synaptic Plasticity

In the brain, various mRNAs are regulated by specific AS-lncRNAs. Recent studies have shown that AS-lncRNAs play a direct role in regulating the expression of genes associated with synaptic plasticity [33]. These genes include those encoding calcium/calmodulin-dependent protein kinase II (CaMKII) and neurogranin (Nrgn) subunits, which are closely related to postsynaptic signaling pathways and long-term potentiation (LTP) [47]. CaMKII has important physiological functions in maintaining long-term synaptic plasticity and plays a role in the early stages of memory consolidation [48]. The overlapping transcripts of both the sense and antisense transcripts of these two proteins enhance the diversity of post-transcriptional regulation of their gene products in cortical development and synaptic functions [47].

Kcna2-AS can regulate the voltage-dependent potassium channel Kcna2, which is associated with human pathological conditions, as mutations in this channel are linked to ataxia and focal epilepsy [49]. Further experiments indicate that the overexpression of Kcna2-AS in models negatively correlates with the expression levels of KCNA2, suggesting that this AS-lncRNA plays a role in regulating proteins associated with synaptic plasticity. In rats, peripheral nerve injury significantly increases the expression of Kcna2-AS through a transcriptional activation mechanism mediated by myeloid zinc finger protein 1 (MZF1), leading to a reduction of approximately 50% in potassium active channels [50]. Experimental results suggest that the induction of Kcna2-AS during nerve injury may reduce voltage-gated potassium currents, thereby increasing the excitability of dorsal root ganglion neurons and resulting in neuropathic pain symptoms.

### 2.1.4. Involvement of AS-lncRNAs in Epigenetic and Transcriptional Regulation

In certain tissues, nuclear lncRNAs act as molecular scaffolds by recruiting chromatin-modifying factors, such as Polycomb Repressive Complex 2 (PRC2) and DNA methyltransferase 3 (DNMT3). This recruitment regulates genomic elements and influences the expression of target genes [51, 52]. In the brain, dynamic global modifications of chromatin are associated with cell lineage commitment, development, terminal differentiation, and complex cognitive functions, such as learning [53-55].

Mutations in the SMN1 gene, which encodes the survival motor neuron protein (SMN), are a primary cause of spinal muscular atrophy (SMA) [56]. In addition to the SMN1 gene, the SMN protein can also be produced from splice variants of the SMN2 gene, with individuals possessing varying numbers of SMN2 genes. The clinical severity of SMA varies based on the SMN2 copy number when an SMN1 mutation occurs [57, 58]. Studies have found that the expression level of the SMN antisense transcript (SMN-AS1) increases during neurogenesis and negatively correlates with SMN protein levels. Researchers have discovered that SMN-AS1 recruits the chromatin modifier PRC2 to the SMN2 promoter, inhibiting its expression. Knockdown of SMN-AS1 causes PRC2 to dissociate from the promoter, resulting in increased total SMN protein levels in neurons. This suggests that SMN-AS1 levels play a crucial role in balancing residual SMN protein, thereby affecting SMA clinical outcomes [58].

The BDNF-AS regulates the expression of the sense strand encoded by brain-derived neurotrophic factor (BDNF). BDNF is a vital secreted growth factor essential for neuronal growth, maturation, differentiation, and maintenance, and its expression is often impaired in neurodegenerative and psychiatric disorders. For instance, BDNF levels are reduced in patients with Huntington's disease (HD). Recent studies indicate that knocking down BDNF-AS results in the upregulation of BDNF levels [23]. BDNF-AS acts through PRC2, which suppresses gene expression primarily by methylating lysine 27 of histone H3 (H3K27me2/3) via its catalytic subunit, enhancer of zeste homolog 2 (EZH2) [26]. Research findings show that after knocking down BDNF-AS, the occupancy of EZH2 and H3K27me3 at the BDNF promoter is reduced [59]. Thus, BDNF-AS plays a crucial role in the development of HD by recruiting EZH2 to the BDNF promoter region, inhibiting BDNF transcription. Additionally, examples of AS-lncRNAs regulating neuronal differentiation and neurogenesis include Hox-encoded HOXA transcript antisense RNA and myeloid-specific 1 (HOTAIRM1). HOTAIRM1 regulates Neurogenin 2, a basic helix-loop-helix transcription factor that serves as a key regulator of neurogenesis [60].

### 2.1.5. Regulation of mRNA Stability, Translation, and Splicing by AS-lncRNAs

AS-lncRNAs can indirectly increase target mRNA expression levels by functioning as molecular sponges or decoys for microRNAs (miRNAs). Disruption of this function can lead to mRNA degradation. For instance, BACE1-AS is an antisense transcript of the BACE1 gene, which encodes the β-secretase 1 (BACE1) protein. BACE1-AS is highly conserved among vertebrates and is essential for producing β-amyloid protein. Its expression levels are elevated in patients with AD and APP transgenic mice [37]. BACE1-AS enhances BACE1 mRNA stability by forming double-stranded RNA with it, thus masking the binding site for miR-485-5p and increasing BACE1 protein levels [61]. Knockdown of this antisense transcript results in decreased BACE1 levels, consequently reducing amyloid formation and aggregation in the brain. Therefore, BACE1-AS is considered a clear biomarker and a potential therapeutic target for AD.

AS-lncRNAs can regulate the translation level of target gene mRNA. UCHL1-AS (ubiquitin carboxyl-terminal hydrolase L1-antisense) can induce Uchl1 translation. Human UCHL1, a neuron-restricted protein, functions as a deubiquitinase, ubiquitin ligase, or monoubiquitin stabilizer. Its inactivation has been reported in patients with AD and Parkinson's disease (PD) [56, 62]. Overexpression of UCHL1-AS increases UCHL1 protein abundance without affecting its mRNA level. This activity depends on a 5' sequence overlapping with UCHL1 and an embedded inverted SINEUP (SINEB2 sequence-regulated translation upregulation). UCHL1-AS enhances Cap-independent UCHL1 protein translation under stress conditions. UCHL1 expression is associated with a delay in AD onset, making UCHL1-AS a crucial regulator of the disease and a promising therapeutic target. When dopaminergic cells were treated with mTOR inhibitors, antisense Uchl1 relocalized to the cytoplasm, binding Uchl1 mRNA to polysomes and increasing UCHL1 protein levels [63]. Since mTOR1 inhibition protects dopaminergic neurons from apoptosis in genetic and neurochemical models of PD, the UCHL1-ncRNA-mTOR1 interaction may be significant for PD development [64].

Another way AS-lncRNAs interfere with cellular function is by regulating RNA splicing. The antisense transcript PINK1-AS is transcribed from the antisense strand of the PINK1 gene (PTEN-induced kinase 1), which is abundant in mitochondrial-rich tissues and frequently mutated in PD [65]. PINK1-AS regulates the expression of the PINK1 splice variant svPINK1 by forming double-stranded RNA that acts in cis. Silencing PINK1-AS decreases svPINK1 expression in neuronal cells [38]. Since svPINK1 encodes the C-terminal isoform of PINK1, this peptide sequence may play a crucial role in disease progression and affect PINK1 kinase activity. Therefore, the regulation of PINK1-AS expression may be directly related to PD.

### 2.2 AS-lncRNAs in Neurodegenerative Disorders

Neuronal damage is highly prevalent in PD, with most symptoms arising from the loss of dopaminergic neurons. Research has shown that certain AS-lncRNAs are involved in the regulation of this damage by modulating autophagy and neuronal apoptosis. For instance, the upregulation of BDNF-AS regulates autophagy and apoptosis in the SH-SY5Y cell model by downregulating miR-125b-5p[66]. Dysregulation of α-synuclein is another common feature of PD, associated with impairments in several cellular processes, including synaptic vesicles, mitochondrial function, and the autophagy-lysosome pathway, ultimately leading to abnormal dopamine levels [67]. Overexpression of OIP5-AS1 reduces α-synuclein accumulation by binding to miR-126 and modulating the PLK2/α-synuclein autophagy pathway [68]. Additionally, MAPT-AS1, which inconsistently regulates the tau-encoding gene MAPT, has been identified as a potential therapeutic target for PD. An in vitro study on a PD cell model demonstrated that MAPT-AS1 exerts inhibitory effects on the MAPT gene through promoter methylation and provides neuroprotective effects similar to those of vitamin E [69]. These findings highlight AS-lncRNAs as potential therapeutic targets for exploring regulatory mechanisms and improving our understanding of Parkinson's disease pathogenesis.

Angelman syndrome is a disorder in which the Ube3a-ATS may be a potential therapeutic target. This neuron-specific transcript, located in the nucleus, regulates the Ube3a gene [70]. Angelman syndrome is a neurodevelopmental disorder characterized by intellectual disability, speech and motor impairments, and often a distinctively happy demeanor [71]. The Ube3a gene encodes a ubiquitin ligase that regulates key neuronal proteins, including MAPK1 and β-catenin, via ubiquitination. Ube3a-ATS typically suppresses the paternal Ube3a allele, and the disease occurs when the maternal allele is deleted. Due to paternal gene imprinting, even if a functional Ube3a copy is present, UBE3A protein expression remains restricted. Thus, downregulating paternal Ube3a-ATS could be an effective therapeutic strategy[72, 73].

Amyotrophic lateral sclerosis (ALS) is a progressive neuromuscular degenerative disease that primarily damages motor neurons in the somatic nervous system. The disease is characterized by the gradual degeneration of neuromuscular junctions, typically starting in distal muscles and ultimately leading to the loss of most motor functions required for daily activities [74]. AS-lncRNAs play regulatory roles in the onset and progression of ALS and exhibit high tissue specificity, making them potential key targets for diagnosis and therapy [75]. Although research on coding transcripts is more advanced, the role of AS-lncRNAs in the pathogenesis of ALS remains in the early exploratory stages. However, recent studies have reported differential expression of certain AS-lncRNAs, which affects transcriptional regulatory pathways. ZEB1-AS1 and ZBTB11-AS1 are novel antisense transcripts associated with ALS [76]. ZEB1-AS1 is an antisense transcriptional regulator of Zinc Finger E-Box Binding Homeobox 1 (ZEB1), a highly conserved transcriptional repressor involved in chromatin and E-box binding. However, compared to healthy controls, ZEB1-AS1 is downregulated in sporadic ALS samples [77]. ZBTB11-AS1 is another differentially expressed antisense transcript associated with the Zinc Finger and BTB Domain Containing 11 gene (ZBTB11), suggesting its potential involvement in transcriptional regulation. In ALS patients, ZBTB11-AS1 is also downregulated [78]. Additionally, other studies have demonstrated the potential role of ATXN2-AS in spinocerebellar ataxia type 2, which is associated with an increased risk of ALS [79].

In summary, AS-lncRNAs may play critical roles in various molecular processes involved in neurodegenerative diseases. Notably, with few exceptions, these AS-lncRNAs are predominantly located near key neuronal sites or oriented in the antisense direction relative to these loci, underscoring the importance of further investigation into these specific molecules. AD, the most common neurodegenerative disorder, will be discussed in detail in the next section, focusing on key AS-lncRNAs identified in AD research.

## 3. Antisense lncRNAs in Alzheimer’s Disease

**Figure 2.**
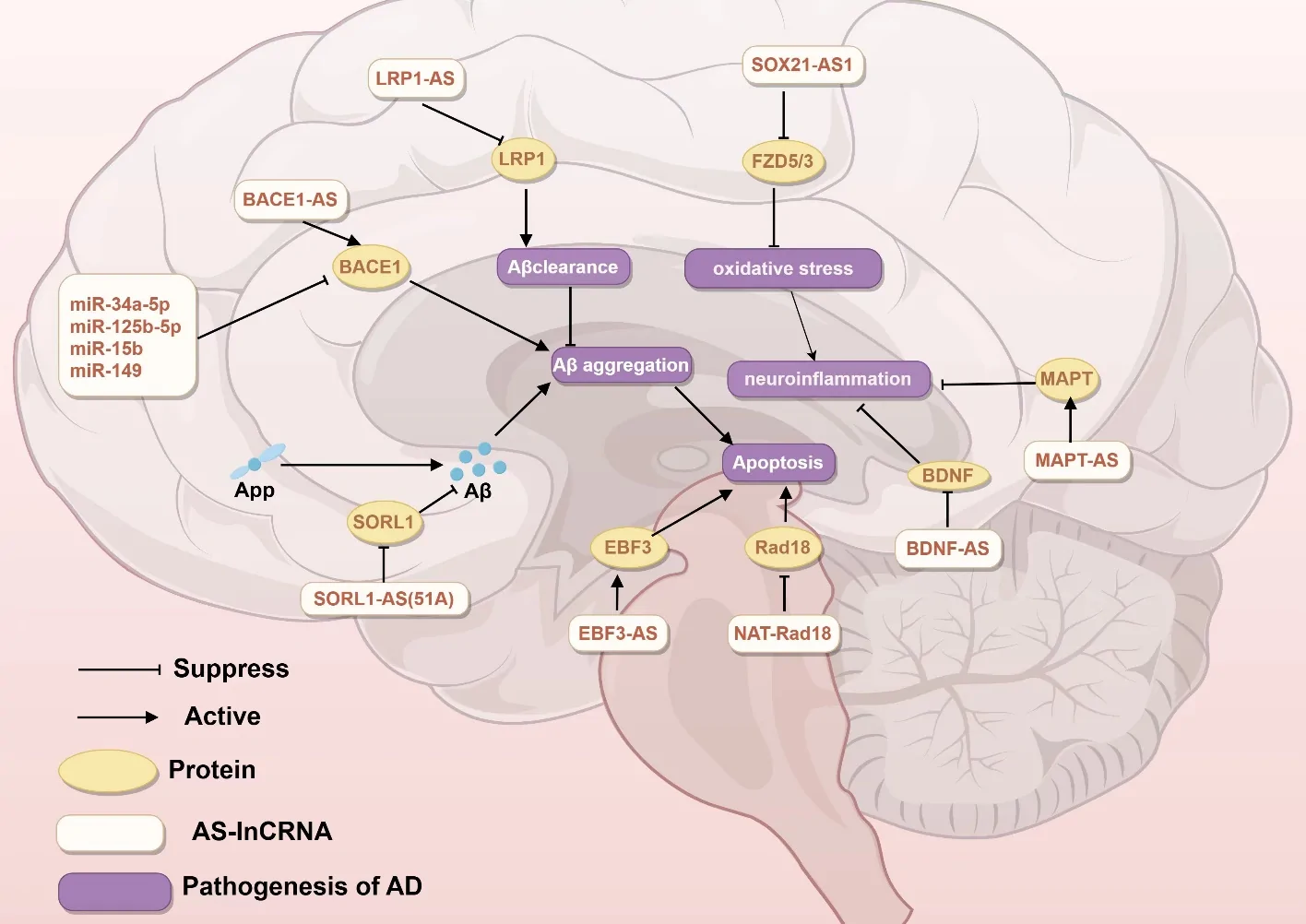
The effects of AS-lncRNAs on pathophysiology in AD.

LncRNAs are crucial regulators of gene expression, exerting influence over various biological processes, including brain aging and neurodegenerative diseases [16]. AS-lncRNAs refer to a class of lncRNAs transcribed from the introns or exons of protein-coding genes on the antisense strand. Studies have indicated that several AS-lncRNAs can modulate processes such as Aβ aggregation, tau phosphorylation, neuronal apoptosis, oxidative stress, neuroinflammation, and synaptic plasticity. These AS-lncRNAs have been shown to play diverse roles in the pathogenesis of AD [80]. In this section, we discuss the role of antisense lncRNA in AD in pathogenesis (Fig. 2).

### 3.1 The regulation of Aβ aggregation and tau protein

#### 3.1.1 BACE1-AS

The pathogenesis of AD involves intricate molecular mechanisms that regulate the expression and activity of key proteins implicated in disease progression. Among these proteins, β-site amyloid precursor protein cleaving enzyme 1 (BACE1) plays a central role in the production of amyloid-beta (Aβ), a peptide associated with AD pathology. BACE1 is a type I transmembrane aspartic protease composed of 501 amino acids, primarily functioning as β-secretase in the amyloidogenic processing pathway [81, 82]. In murine models, BACE1 has been demonstrated to modulate voltage-gated sodium channels, which regulate neuronal activity involved in AD pathophysiology. Notably, BACE1 expression localizes to the presynaptic terminals surrounding amyloid plaques, suggesting that BACE1-deficient mice exhibit a healthier phenotype and suppress the production of β-amyloid [83]. The dysregulation of BACE1 plays a pivotal role in initiating toxic processes in the early stages of the disease [76]. Several microRNAs, including miR-34a-5p, miR-125b-5p, miR-15b, and miR-149, have been shown to suppress BACE1 expression, thereby reducing Aβ accumulation and improving neuronal damage [83].

BACE1-AS is a conserved RNA transcribed from the opposite strand of the BACE1 gene locus on chromosome 11q23.3 [37, 84]. BACE1-AS regulates BACE1 mRNA and subsequent protein expression both in vitro and in vivo [85]. Increased expression of BACE1-AS has been observed in the blood and brain of AD patients, as well as in AD animal models, promoting AD progression through its effects on BACE1 activity. Studies have also shown that knocking out BACE1-AS can reduce BACE1 and Aβ levels, inhibit tau phosphorylation in the hippocampus, and improve learning and memory in AD mice [84, 86]. Upregulation of BACE1-AS leads to elevated levels of Aβ42 in AD brain tissue and mouse models [87]. BACE1-AS competes with miR-485-5p to regulate BACE1-1 competitively, and the dysregulation of both is associated with increased BACE1 expression in AD brain slices [61]. Additionally, BACE1-AS is also subject to positive regulation [37]. Transcripts binding to BACE1 mRNA promote BACE1 expression [88].

LncRNA BACE1 and BACE1-AS are highly expressed in the blood of brain-related diseases. They participate in the pathogenesis of AD by increasing Aβ and APP levels and are mainly involved in learning and memory impairments through the stabilization of neuronal RNA-binding protein HuD during AD progression [88]. The pathogenesis of AD is associated with various cellular stressors. Exposure to high temperatures, serum starvation, streptozotocin, Aβ1-42, high glucose, BACE1-AS, and BACE1 mRNA upregulate BACE1-AS expression, suggesting that the increase in BACE1-AS expression may be associated with cellular stressors that drive the upregulation of BACE1 mRNA and protein levels, thereby promoting the biosynthesis of Aβ1-42 in the human AD brain [37]. Whether BACE1-AS is knocked down or overexpressed, both BACE1 mRNA and BACE1 protein are concomitantly regulated, resulting in decreased production and deposition of Aβ [37, 84, 89]. BACE1 deficiency in animals leads to various behavioral and physiological defects, including memory decline, reduced synaptic plasticity, emotional defects, and peripheral myelin sheath formation defects [90-96]. The subtle physiological and pathological boundaries indicate that the expression of BACE1 should be strictly regulated [37, 96].

In summary, cellular stress increases the levels of BACE1-AS, thereby stimulating BACE1 expression and enhancing the processing of APP and the production of Aβ1-42. Elevated levels of Aβ1-42 further promote overexpression and feedforward APP processing cascade reactions [37, 89, 97]. By forming RNA duplexes, BACE1-AS enhances the stability of BACE1 mRNA [89, 98, 99]. Therefore, BACE1 and BACE1-AS may serve as potential biomarkers and therapeutic targets for AD [93, 97, 100, 101].

#### 3.1.2 SORL1-AS(51A)

Sortilin-related receptor L1 (SORL1), commonly referred to as SORLA or LR11, is located on human chromosome 11q23.2-q24.2. Its encoded protein SORL1 is a transmembrane neuronal sorting protein with unknown function, specifically and abundantly expressed in neurons [102-104]. SORL1 is a multifunctional endocytic receptor involved in APP trafficking and is a potential genetic predisposing factor for AD [105, 106]. The SORL1 gene encodes Sortilin-related receptor 1 (SORLA), which can block the production of amyloid-β (Aβ) [107]. Katrin et al. found a significant decrease in SORL1 expression levels in cortical and hippocampal neurons of AD patients [108, 109]. Study also reported reduced expression of SORL1 in the cerebrospinal fluid (CSF) of AD patients [110]. Furthermore, BACE1 activity detected in the CSF showed a positive correlation with SORL1 concentration [111]. These findings suggest a potential role for SORL1 in the pathogenesis of AD.

SORL1-AS (51A) is the antisense orientation of SORL1 intron 1, generated by selective splicing. Upregulation of SORL1-AS leads to reduced expression of SORL1 by altering mRNA splicing, thereby impairing APP processing [112]. Mechanistically, lncRNA 51A binds to the splice site of SORL1 precursor mRNA through base pairing, resulting in splice site displacement and decreased expression of the typical variant A [112]. Therefore, the protective gene SORL1 and lncRNA 51A may play important roles in AD by inhibiting the expression of the typical variant A of SORL1. Research by Gómez-Tortosa et al. suggests that blocking SORL1 can promote amyloidogenesis and greatly increase the risk of developing AD [113]. It was also found that 51A is significantly upregulated in the human brain, especially in the cerebral cortex of AD patients, indicating a potential biological therapeutic target for AD [114].

#### 3.1.3 LRP1-AS

Low-density lipoprotein receptor-related protein 1 (LRP1) belongs to the low-density lipoprotein receptor family, and it plays a role in various physiological processes, including cellular transport of cholesterol, ligand endocytosis, and transcytosis across the blood-brain barrier (BBB) [115]. LRP1 is abundantly expressed in different cell types in the brain, including neurons, vasculature, and glial cells [116, 117]. LRP1 is crucial for Aβ clearance as it mediates the uptake and degradation of Aβ in astrocytes, microglia, and neurons [118, 119]. It coordinates the migration of Aβ across the BBB via brain vascular smooth muscle cells together with ABCB1/P-glycoprotein (P-gp) [120]. LRP1 not only promotes Aβ clearance through endocytosis but also binds to APP on the cell surface, facilitating the endocytic trafficking of APP and thereby increasing amyloidogenic processing [121]. Additionally, the C-terminal transmembrane domain of LRP1 reduces Aβ production by competing with β-secretase and γ-secretase cleavage sites on APP [122]. Thus, LRP1 plays a critical dual role in the production and clearance of Aβ.

LRP1-AS is a 1387 nt long non-coding RNA, which is a natural antisense transcript of low-density lipoprotein receptor-related protein 1 (LRP1), transcribed from the opposite strand of the LRP1 gene [123]. Upregulation of LRP1-AS in the AD brain is associated with promoting Aβ formation and reducing clearance [123, 124]. LRP1-AS negatively regulates the expression of Lrp1 directly or indirectly at the RNA and protein levels [123]. LRP1-AS directly binds to high-mobility group box 2 (Hmgb2) and inhibits Hmgb2-enhanced Srebp1a transcriptional activity on LRP1, thereby disrupting LRP1-mediated Aβ clearance [123]. Short oligonucleotides of LRP1-AS inhibit the antisense transcript-Hmgb2 protein interaction by enhancing Hmgb2 activity and enhancing LRP1 expression [123]. These findings suggest that LRP1-AS may epigenetically downregulate LRP1 expression in the brains of AD subjects, impairing Aβ clearance and leading to amyloid plaque aggregation.

#### 3.1.4 MAPT-AS1

The microtubule-associated protein tau (MAPT) gene is encoded by a single copy gene located on chromosome 17q21.21 and is expressed as eight isoforms through selective mRNA splicing [125]. Upregulation of MAPT leads to an increase in events associated with neurodegeneration [126]. MAPT-AS1 is an 840 bp RNA transcribed from the opposite strand of the MAPT locus (17q21.31), composed of two exons [127]. The molecular localization of MAPT-AS1 is predominantly in the cytoplasm compared to the nucleus, and MAPT and MAPT-AS1 may form RNA-RNA duplex structures, protecting them from degradation by RNases [127]. Recent studies have shown that delivering MAPT-AS1 vectors via adenovirus to the hippocampus of mouse models can result in reduced tau levels [128]. These findings suggest that MAPT-AS1 represents a potential therapeutic approach for treating AD [129].

### 3.2 Neuroinflammation and Synaptic Plasticity

#### 3.2.1 MAGI2-AS3

Membrane-associated guanylate kinase inverted 2 (MAGI2) is a novel lncRNA transcribed from chromosome 7q21.11. Its expression is typically concentrated in the nucleus of SK-N-SH cells [130, 131]. MAGI2-AS3 is a single-copy RNA located on chromosome 7 and is the antisense transcript of the MAGI2 gene. It has been reported to participate in the suppression of various tumors, such as bladder cancer, glioma, non-small cell lung cancer, ovarian cancer, and breast cancer [131]. Compared to healthy controls, the expression of MAGI2-AS3 is significantly upregulated in the serum of AD patients, while the level of miR-374b-5p is downregulated. Additionally, the expression level of MAGI2-AS3 is positively correlated with the severity of the disease in AD patients, whereas miR-374b-5p shows the opposite trend [132, 133]. In AD cell models, MAGI2-AS3 can regulate MAGI2 mRNA levels, Aβ-induced neurotoxicity, and neuroinflammation through miR-374b-5p, suggesting that MAGI2-AS3 may be a potential therapeutic target for AD [134, 135].

#### 3.2.2 BDNF-AS

Brain-derived neurotrophic factor (BDNF) is a member of the neurotrophin family of growth factors, playing crucial roles in neuronal development and survival, neurite growth and differentiation, synaptic plasticity, and neurotransmitter release [136-139]. Previous studies have indicated that the decreased levels of BDNF mRNA and protein in serum and the human brain are closely associated with the pathogenesis of AD [140-142]. Multiple lines of evidence suggest that amyloid-beta (Aβ) can induce cognitive impairment and memory loss by downregulating BDNF expression [143-145]. Aβ reduces BDNF levels by decreasing the phosphorylation of cAMP response element-binding protein (CREB). BDNF pretreatment in primary cultured neurons has shown potential protective effects against Aβ-induced neurotoxicity [146]. Therefore, BDNF plays a crucial role in Aβ-induced synaptic damage in neurons and cognitive impairment in AD patients.

The BDNF locus is located on chromosome 11 and exhibits transcriptional activity on both strands, leading to the transcription of non-coding NATs [147]. BDNF-AS is a conserved non-coding antisense RNA transcript of BDNF, which dynamically regulates the expression of BDNF mRNA and protein both in vitro and in vivo [23, 148]. BDNF-AS suppresses BDNF transcription by modulating the H3K27me3 at the BDNF locus and recruiting EZH2 to the BDNF promoter region[23]. Blocking BDNF-AS leads to increased expression of Bdnf mRNA and protein in vivo. Previous studies have found that Aβ25-35 significantly increases the levels of BDNF-AS in PC12 cells and decreases BDNF levels, accompanied by a reduction in PC12 cell viability and induction of apoptosis [149]. However, silencing BDNF-AS significantly upregulates Aβ25-35-induced BDNF reduction, increases cell viability, and inhibits PC12 cell apoptosis [149]. These findings suggest that inhibition of BDNF-AS is a promising strategy for treating AD, particularly by increasing BDNF levels.

### 3.3 Regulation of Neuronal Apoptosis

#### 3.3.1 EBF3-AS

Early B cell factor 3 antisense RNA (EBF3-AS) is a long non-coding RNA containing two exons of 842 nt in length, transcribed from the antisense strand of the protein-coding gene Early B Cell Factor 3 (EBF3) on chromosome 10 [132]. RNA sequencing revealed significant and abundant differences in the expression of EBF3-AS in the brains of late-onset AD patients compared to controls [95]. Additionally, the expression levels of EBF3-AS were higher in the hippocampus of APP/PS1 transgenic rats than in normal rats [132]. This suggests that EBF3-AS may play a crucial role in AD. Knockdown of EBF3-AS with siRNA inhibited cell apoptosis induced by Aβ25-35 and okadaic acid (OA) in an AD mouse model, both of which increased the expression of EBF3 [150]. They observed that lncRNA EBF3-AS stimulated EBF3 expression and promoted neuronal apoptosis in the brains of AD mice [150]. These results suggest that lncRNA EBF3-AS promotes neuronal apoptosis in AD and is involved in regulating the expression of EBF3. EBF3-AS may represent a novel therapeutic target for the treatment of AD.

#### 3.3.2 NAT-RAd18

Cell apoptosis is the primary form of programmed cell death, and excessive apoptosis can lead to progressive cell loss, resulting in various neurodegenerative diseases, including AD. Rad18 serves as a major PCNA-directed E3 ubiquitin ligase. In S-phase cells, the loss of Rad18-mediated DNA damage tolerance mechanism can result in sustained transduction of DNA damage signals through ATR/Chk1 [151, 152]. NAT-Rad18, the gene encoding the natural antisense transcript of Rad18, encodes a series of DNA damage factors and participates in the regulation of cell apoptosis [132]. There exists a balanced relationship between Rad18 and NAT-Rad18 at both the mRNA and protein levels, with Rad18 expression levels being relatively low. NAT-Rad18 is expressed in brain tissue after exposure to Aβ, particularly in cortical neurons. Following treatment with Aβ40, upregulated NAT-Rad18 promotes DNA damage and cortical neuron death by reducing Rad18 expression[153]. Rad18 is recruited to the chromatin compartment under conditions of DNA damage[154]. NAT-Rad18 silences Rad18, which functions to balance the expression levels of target genes. Its upregulation is associated with Aβ-derived neurotoxicity and cell apoptosis [155]. In summary, these results suggest that NAT-Rad18 may participate in the development of AD through its function in the DNA repair system, providing new insights into potential therapeutic targets for AD.

### 3.4 The Impact of Oxidative Stress

#### 3.4.1 SOX21-AS1

Sox2 is a component of the core transcriptional regulatory network critical for maintaining stem cell pluripotency and neurogenesis [156]. Previous studies have identified Sox2 Overlapping Transcript (Sox2OT) as an evolutionarily conserved transcript in vertebrates, associated with cognitive impairment in AD mouse models [157, 158]. Sox2OT is expressed in the developing mouse brain cortex, promoting neuronal differentiation and neurogenesis by suppressing Sox2 in neural progenitor cells [159]. Additionally, whole-genome microarray analysis indicates differential expression of Sox2OT in early and late stages of disease in AD model mice, suggesting its potential relevance to AD pathobiology [160]. SRY-Box 21 Antisense RNA 1 (SOX21-AS1) is a 2986 bp lncRNA sharing bidirectional promoter with SOX21 on human chromosome 13q32.1 [161]. SOX21-AS1 has been described as a favorable prognostic biomarker for cervical and oral cancers [161, 162]. Aberrant expression of SOX21-AS1 has been observed in the nervous system [163], and microarray analysis of AD chips (GSE4757) revealed upregulation of SOX21-AS1 in AD, suggesting its potential role in AD occurrence and progression[164]. Frizzled 3/5 (FZD3/5) are important receptors in the Wnt signaling pathway involved in central nervous system development. Silencing SOX21-AS1 upregulates FZD3/5, subsequently activating the Wnt signaling pathway, reducing neuronal oxidative stress, preventing neuronal apoptosis in AD mice, and ultimately improving learning and memory in AD [164]. Additionally, the action of SOX21-AS1 is similar to NEAT1, both acting on miR-107, which is found dysregulated in brain tissue of AD patients. Specifically, knockdown of SOX21-AS1 attenuates neuronal apoptosis and alleviates oxidative stress in AD by sponging miR-107, thereby reducing Aβ-induced neuronal damage [165, 166]. These results suggest that SOX21-AS1 may participate in AD development through oxidative stress and could serve as a novel therapeutic target for AD.

## 4. Diagnostic Potential of Antisense lncRNAs

The classical diagnosis of AD relies on neuroimaging techniques and biomarkers in plasma or cerebrospinal fluid, which support clinical manifestations of the disease [167]. However, research indicates that the neuropathological mechanisms of the disease begin decades before clinical symptoms appear [168]. Therefore, establishing a biological definition of AD still depends on biomarkers that can reflect biological changes in the early stages of the disease [169]. As previously mentioned, AS-lncRNAs have been identified as crucial regulatory factors closely associated with the onset and progression of AD.

Specifically, the antisense lncRNA BACE1-AS in plasma has shown potential as a diagnostic biomarker for AD. Studies have found that BACE1-AS levels in the plasma of AD patients are significantly higher than those in healthy controls, and its levels correlate with the severity of cognitive impairment in AD patients [170]. Further research indicates that the expression differences of BACE1-AS are more pronounced when patients are divided into preclinical AD and full AD stages, suggesting its potential in early diagnosis. ROC curve analysis shows that BACE1-AS can effectively distinguish between pre-AD and full-AD groups, demonstrating its efficacy as an AD biomarker [171]. Another study explored changes in BACE1-AS within plasma exosomes, finding that its levels remained elevated in AD patients, with a sensitivity of 87.5% and a specificity of 61.3% [172]. However, other studies have found that BACE1-AS levels are lower in the preclinical stage of AD and significantly higher in the full stage of the disease. This finding highlights the challenges of early diagnosis and emphasizes the importance of considering disease staging in research design [171].

Additionally, another widely studied AS-lncRNA is SORL1-AS (51A), which is also associated with the pathogenesis of AD [108, 173]. SORL1-AS expression leads to the accumulation of Aβ-42 [174]. Research has shown that levels of SORL1-AS in plasma and brain are increased in AD patients compared to controls and are negatively correlated with the Mini-Mental State Examination (MMSE) scores of AD patients, indicating its potential as an important diagnostic biomarker for AD [175, 176].

However, it is important to emphasize that despite the promising and helpful findings, relying on circulating AS-lncRNAs as biomarkers for brain diseases presents challenges. While qRT-PCR can be used to detect the expression of AS-lncRNAs in the bloodstream, there are currently no known standard references for AS-lncRNAs from different sources [177]. Additionally, most AS-lncRNAs have low expression levels, making them difficult to detect. Importantly, AS-lncRNAs may be dysregulated in multiple diseases rather than being specific to a single disease [178]. Therefore, liquid biopsies should be used in conjunction with existing diagnostic methods rather than relied upon solely [177]. Currently, cerebrospinal fluid biomarkers are still considered superior to plasma biomarkers, as cerebrospinal fluid directly contacts the brain's interstitial fluid, potentially providing a more accurate reflection of metabolic and pathological changes in the brain. Some exosomal miRNAs derived from cerebrospinal fluid have been validated as biomarkers [179]. However, there remains a lack of in-depth research on AS-lncRNAs and their potential roles in AD diagnosis.

In conclusion, while the data are promising and the potential of AS-lncRNAs as diagnostic tools is noteworthy, more research is needed before AS-lncRNAs-based AD diagnosis can be clinically applied. Additionally, the combination of AS-lncRNAs with other circulating biomarkers and the morphological and physical characteristics of brain tissue should be considered to enhance the reliability of diagnostic results [172].

## 5. Therapeutic Perspectives and Challenges

AS-lncRNAs play a crucial role in the pathogenesis and progression of AD. Therefore, their regulation may represent promising tools for AD therapy. In recent years, significant progress has been made in the development of biologic drugs targeting RNA molecules within cells, laying the foundation for lncRNA-based AD therapies. Small interfering RNA (siRNA) can be used to inhibit AS-lncRNAs in cells. Additionally, considerable efforts have been devoted to developing siRNA-based therapies for various metabolic disorders, cancers, neurodegenerative diseases, and conducting in vitro and in vivo studies of single-gene function [180, 181]. In 2018, the world's first siRNA-based drug, Patisiran, was approved for the treatment of hereditary transthyretin-mediated amyloidosis[182], and another siRNA-based drug, Givosiran, was approved in 2020 for adults with acute hepatic porphyria [183]. Despite the therapeutic potential of siRNA being evaluated in different stages of clinical trials, the major challenges remain the half-life and delivery of siRNA.

Antisense oligonucleotides (ASOs) are small single-stranded nucleic acids that can target AS-lncRNAs in both the nucleus and cytoplasm. ASO-mediated knockdown of lncRNA has been shown to have therapeutic efficacy in patients with Angelman syndrome, a single-gene disorder characterized by intellectual disability [72, 184]. Significant clinical breakthroughs have been made in ASO-related treatments over the past decade. Several ASO drugs have been approved by the FDA for the treatment of neurodegenerative diseases [185]. One ASO drug, nusinersen, has been approved for the treatment of spinal muscular atrophy. Importantly, when administered to symptomatic patients, nusinersen not only improves disease symptoms but also slows disease progression. Building on the early success of nusinersen, ASOs hold significant promise for treating neurological diseases [186]. Furthermore, ASOs have shown promising efficacy in targeting Huntington's gene (HTT) for Huntington's disease (HD), targeting SOD1 and C9ORF72 for amyotrophic lateral sclerosis (ALS), and targeting MAPT (TAU) for AD [187]. The ASO-mediated lncRNA knockdown approach may represent an innovative therapeutic strategy for treating AD. By developing specific ASOs to downregulate lncRNAs, it may be possible to reduce Aβ accumulation in AD, which has been demonstrated to be effective in AD cell models and animal models [84, 89, 123, 188, 189]. Therefore, further exploration of the molecular mechanisms and complex interaction networks of lncRNAs to target lncRNAs regulating Aβ homeostasis is needed as a novel therapeutic strategy for AD.

The CRISPR/Cas9 system is an adaptive immune defense mechanism that has evolved in prokaryotic organisms such as bacteria and archaea [8]. This system consists of a single-guide RNA (sgRNA) and the Cas9 enzyme; the sgRNA directs the Cas9 nuclease to a specific genomic site through complementary base pairing, enabling Cas9 to cleave the DNA sequence. Due to its exceptional accuracy, efficiency, durability, and programmability, CRISPR/Cas9 has been effectively utilized to target lncRNAs, offering new insights into lncRNA research [190, 191]. It has facilitated the exploration of lncRNA functions as well as their screening and annotation [191]. Studies have shown that knocking down LINC00341 using CRISPR-CasRX can inhibit the growth of bladder cancer cells, induce apoptosis, and suppress their activity [192]. Additionally, research indicates that CRISPR interference (CRISPRi) technology can regulate lncRNA to treat oligozoospermia caused by varicocele [193]. Despite these advances, the clinical application of lncRNA-targeted CRISPR/Cas9 therapies faces significant challenges. Similar to siRNA and ASO, the CRISPR/Cas9 system also suffers from off-target effects [8]. Current approaches for addressing these issues include off-target prediction, detection, and prevention, although no completely effective solution has been established [194]. Various algorithms and technologies have been developed to predict and detect off-target effects, including biophysical models based on the kinetics of sequence-target recognition and whole-genome off-target analysis techniques that identify off-target mutations through whole-genome sequencing, minimizing interference from single nucleotide variants [195-197]. Furthermore, off-target effects can be mitigated by modifying the design of Cas9 or sgRNA and by truncating the sgRNA [198]. However, designing highly specific sgRNAs for lncRNAs remains a key challenge. Nevertheless, as technology advances and methodologies improve, the potential of CRISPR/Cas9 in clinical applications continues to be explored and expanded. Addressing off-target effects and other technical challenges is essential for the successful clinical application of this technology, particularly in treating human diseases [4, 199].

Despite the promising outlook, there are concerns about the potential adverse effects of gene therapies targeting lncRNA compared to traditional drug targets and proteins. The primary risk lies in the insufficient fundamental research on the functions and potential downstream effects of druggable lncRNAs. Consequently, the clinical use of lncRNA-targeting drugs might result in unforeseen risks and inappropriate pathological effects. Additionally, germline gene therapy, which results in genetic modifications that can be inherited by future generations, is a subject of intense debate [200]. A recent study in China announced that the genomes of twins were edited using CRISPR/Cas9 during in vitro fertilization, prompting widespread condemnation for prematurely deploying an experimental biomedical technology in a clinical setting [201]. Ethical considerations and patient acceptability are critical aspects of gene therapy that must be carefully addressed to ensure the responsible development of these advanced treatments [202].

Overall, the above studies demonstrate the tremendous potential of AS-lncRNAs as molecular biomarkers for AD and as targets for AD therapy, encouraging further research to deepen our understanding of the molecular mechanisms and evaluate prospective novel therapeutic targets.

## 6. Future Directions in Research and Therapy

AS-lncRNAs play multifaceted roles in the regulation of RNA networks, which are essential for biological processes [203]. Recent studies have reported dysregulation of AS-lncRNAs in various human diseases, including neurodegenerative disorders. With advancements in research technologies, many AS-lncRNAs involved in the pathophysiology of AD have been identified, providing new insights into their roles in this condition [22]. Targeting AS-lncRNAs presents several advantages for treating AD. First, most AS-lncRNAs exhibit high target specificity, as they regulate their sense genes in a cis manner, ensuring minimal off-target effects. Furthermore, targeting AS-lncRNAs is more aligned with mimicking the natural cellular environment compared to targeting protein-coding genes [203]. For instance, directly inhibiting BACE1 may disrupt the functionality of its protein products, leading to adverse side effects [204, 205]. In contrast, targeting BACE1-AS not only reduces BACE1 levels but also diminishes the abnormal Aβ deposition associated with AD [171].

However, several limitations and challenges must be addressed before fully exploiting the therapeutic potential of AS-lncRNAs. For example, various AS-lncRNAs are involved in the trans-regulation of numerous genes. While targeting AS-lncRNAs may restore optimal expression levels of specific genes, it could simultaneously cause dysregulation of others. Additionally, many AS-lncRNAs share overlapping sequences with their sense counterparts, making it crucial to ensure that the molecules used for targeting do not inadvertently affect these equivalents [206]. Although we have begun to elucidate the functions of some AD-related AS-lncRNAs, our understanding of their molecular mechanisms remains limited, hindering our ability to identify clinically translatable therapeutic targets and develop effective strategies. Integrating research on AS-lncRNAs with existing transcriptomics, proteomics, metabolomics, and genomics data can provide a comprehensive understanding of disease mechanisms. This integration aids in identifying new biomarkers and therapeutic targets, laying the groundwork for personalized treatment strategies. Moreover, combining data from different biological layers can reveal critical aspects of disease progression and facilitate the development of more effective interventions.

The complexity of AD necessitates a multidisciplinary research approach. By combining genetics, neurobiology, and pharmacology, researchers can gain a more holistic understanding of disease mechanisms. Genetics can uncover how genetic variations influence disease risk, neurobiology examines the disease's impact on brain function, and pharmacology focuses on developing new drugs and therapeutic methods. Through collaboration among these disciplines, researchers can better identify the disease's pathological processes and formulate more effective treatment strategies. This integrated approach will significantly advance AD research and treatment.

**Table 1:** AS-lncRNA dysregulation in AD.

AS-lncRNA	Expression	Targets	Regulations	Effects	Ref(s)
**BACE1-AS**	up	Upregulating BACE1 mRNA stability	Modulate mRNA stability	Aβ ↑	[82, 87, 89, 99]
**SORL1-AS (51A)**	up	Downregulating SORL1 variant A	Modulate mRNA splicing processing	Aβ ↑	[106, 113]
**LRP1-AS**	up	negatively regulates the expression of LRP1 directly or indirectly at the RNA and protein levels	Regulate mRNA expression	Aβ ↑, amyloid plaque aggregation	[123]
**MAPT-AS1**	down	Forming an RNA double-stranded structure, Upregulating MAPT mRNA stability	Modulate mRNA stability	Tau ↓	[126, 127, 129]
**MAGI2-AS3**	up	Regulating miR-374b-5p	Modulate mRNA expression	Aβ ↑	[133, 135]
**BDNF-AS**	up	Regulation of H3K27me3 at the BDNF site and recruitment of EZH2 to the BDNF promoter region inhibit BDNF transcription	Regulate mRNA and protein expression	Neuronal apoptosis ↑	[23, 148]
**EBF3-AS**	up	Stimulating the expression of EBF3	Modulate mRNA expression	Neuronal apoptosis ↑	[133, 150]
**NAT-RAd18**	up	Controlling the expression of DNA repair protein Rad18	Regulate the DNA repair system	DNA damage and cortical neuron death ↑	[153, 155]
**SOX21-AS1**	up	Upregulating FZD3/5, activating Wnt signaling pathway and sponging miR-107	Modulate miRNA stability	Neuronal apoptosis and oxidative stress ↑	[164, 166]

AD: Alzheimer's disease; Aβ:amyloid β peptide;BACE1:β-site amyloid precursor protein cleaving enzyme 1;BACE1-AS: antisense transcript of the BACE1 gene ;Aβ: amyloid-beta; SORL1:Sortilin-related receptor L1;SORL1-AS (51A): antisense transcript of the SORL1 gene;LRP1:Low-density lipoprotein receptor-related protein 1;LRP1-AS:antisense transcript of the LRP1 gene; MAPT: Microtubule-associated protein tau ; MAPT-AS1:antisense transcript of the MAPT gene; MAGI2: Membrane-associated guanylate kinase inverted 2;MAGI2-AS3:antisense transcript of the MAGI2 gene; BDNF: Brain-derived neurotrophic factor; BDNF-AS :antisense transcript of the BDNF gene;EBF3:Early B cell factor 3 antisense RNA;EBF3-AS:antisense transcript of the EBF3 gene;NAT-RAD18:antisense transcript of Rad18;SOX21-AS1: antisense transcript of the SOX21 gene

## 7. Conclusion

As the most common type of dementia, AD presents a significant global challenge. In recent years, with advancements in sequencing technologies, numerous differentially expressed AS-lncRNAs associated with AD have been discovered. Although the roles of AS-lncRNAs are complex, research on relative AS-lncRNAs in AD remains limited. However, some AS-lncRNAs are considered potential biomarkers and therapeutic targets for AD. Indeed, certain AS-lncRNAs have been shown to play roles in AD pathology, including BACE1-AS, SORL1-AS(51A), LRP1-AS, MAPT-AS1, BDNF-AS, MAGI2-AS3, EBF3-AS, NAT-RAd18D, and SOX21-AS1. AS-lncRNAs regulate the expression of AD-related genes through various mechanisms to play a key role in Aβ production, clearance, and its induced neurotoxicity (Table 1). While much of this research has focused on the impact of AS-lncRNAs on Aβ aggregation, tau phosphorylation, synaptic plasticity, and neuroinflammation in AD, further in-depth and insightful investigations are warranted.

However, there are still many challenges to overcome in this field. Despite the identification of numerous AS-lncRNAs associated with AD, functional characterization of most AS-lncRNAs remains lacking. It is a daunting task to identify functionally important AS-lncRNAs from the multitude of annotated non-coding transcripts. Moreover, while we have begun to elucidate the functions of some AD-related lncRNAs, our understanding of their molecular mechanisms is limited. All of these factors pose significant limitations in identifying therapeutic targets with translational potential and developing targeted therapeutic strategies. Furthermore, as AS-lncRNAs emerge as potential biomarkers and novel therapeutic targets for AD treatment, the next challenge will be piecing together the entire RNA regulatory network of AS-lncRNAs, assigning them functions in physiological and pathological contexts, and identifying prospective novel therapeutic targets and innovative diagnostic tools.
